# Outcomes of Draining Seton Insertion Followed by Ligation of the Intersphincteric Fistula Tract Versus Cutting Seton Insertion for Anal Fistula: A 5-Year Retrospective Cohort Study

**DOI:** 10.34172/mejdd.2025.442

**Published:** 2025-10-31

**Authors:** Siavash Masoumi, Ahmed Mohammed Ali Hussein Alhurry, Seyed Vahid Hosseini, Sara Shojaei-Zarghani, Mohammad Mostafa Safarpour, Zahra Ghanbarzadegan, Alimohammad Bananzadeh

**Affiliations:** ^1^Colorectal Research Center, Shiraz University of Medical Sciences, Shiraz, Iran; ^2^School of Medicine, Shiraz University of Medical Sciences, Shiraz, Iran; ^3^Department of General Surgery, Imam Hussein Medical City, Karbala, Iraq; ^4^Laparoscopy Research Center, Shiraz University of Medical Sciences, Shiraz, Iran

**Keywords:** Rectal fistula, Seton insertion, Ligation of the intersphincteric fistula tract, Recurrence, Fecal incontinence, Quality of life

## Abstract

**Background:**

This study aimed to compare healing time, recurrence rates, incidence of incontinence, and quality of life between the ligation of the intersphincteric fistula tract (LIFT) procedure (preceded by draining seton insertion) and cutting seton insertion for anal fistula over a 5-year period.

**Methods:**

A retrospective cohort study was conducted on patients with high-type perianal fistulas who underwent either LIFT (preceded by draining seton insertion) or cutting seton insertion between May 16, 2018, and April 17, 2024, at Shahid Faghihi and Ghadir mother and child hospitals. A comparative analysis was performed to evaluate healing time, recurrence rates, incidence of incontinence, and quality of life between the two surgical approaches.

**Results:**

The study included 51 patients who underwent LIFT surgery and 48 who received cutting seton insertion. There were no significant differences between the two groups in terms of demographic characteristics or behavioral factors. Additionally, no statistically significant differences were observed between the two groups regarding fecal incontinence, healing rates, recurrence, healing time, and quality of life (*P*>0.05). However, patients who underwent cutting seton insertion exhibited a non-significant lower recurrence rate compared with those who received the LIFT procedure (8.3% vs. 17.6%).

**Conclusion:**

This study provides valuable insights into the comparison of the efficacy of cutting seton insertion versus the LIFT procedure for managing anal fistulas. By evaluating key postoperative outcomes, the findings may help in clinical decision-making and enhance patient care strategies in colorectal surgery.

## Introduction

 A fistula is an abnormal, epithelialized connection between two epithelial surfaces. An anal fistula, or fistula-in-ano, connects the anorectum to the perianal skin. It is one of the most common surgical conditions, with an incidence rate of up to 2.8 cases per 10,000 individuals.^1^

 Approximately 30%–35% of anorectal abscesses progress to anal fistulas. This rate is notably higher in non-diabetic patients and those under the age of 40, with no significant differences observed based on sex, smoking status, human immunodeficiency virus (HIV) status, or the use of perioperative antibiotics. Patients with fistula-in-ano often present with a range of symptoms, including a history of anorectal abscesses with spontaneous or surgical drainage, external bumps that become irritated and bleed, chronic external drainage, and cyclical perianal pain and swelling that are relieved by the expression of fluid.^2^ Discharge from a perianal opening is the most common symptom, particularly among young men. If left untreated, anal fistulas can lead to fecal incontinence, significantly impacting a patient’s quality of life.^3^ Fistulas are classified based on their anatomical location and relationship to the anal sphincter complex into several types: intersphincteric, trans-sphincteric, suprasphincteric, extrasphincteric, and supralevator fistulas. A complex anal fistula is defined as a trans-sphincteric fistula involving more than 30% of the sphincter complex. Fistulas associated with chronic diarrhea, inflammatory bowel disease, radiation, malignancy, or pre-existing fecal incontinence are also classified as complex.^4^

 The primary goals in treating anal fistulas are to close the internal opening of the fistula tract, drain any infection or necrotic tissue, and eliminate the fistulous tract while preserving sphincter function.^5^ The management of these fistulas presents a significant challenge due to the delicate nature of the anal sphincter musculature and the risk of postoperative complications, such as incontinence and recurrence. Sphincter-preserving procedures, such as ligation of the intersphincteric fistula tract (LIFT), fistula plug repair (FPR), and advancement flaps are often preferred for complex or high-type fistulas.^6^ The LIFT procedure has emerged as a less invasive option that minimizes damage to the anal sphincter. It involves identifying and ligating the fistula tract within the intersphincteric space, promoting healing while preserving sphincter function. The LIFT procedure is associated with favorable healing rates and low complication rates.^7^

 A seton is a string-like material threaded through the fistula tract to induce an inflammatory response, promoting fibrosis. This process helps stabilize the tract, facilitate controlled cutting, and prevent sphincter retraction.^8^ There are three types of setons: cutting, draining, and medicated. The cutting seton has been widely used to manage complex fistulas, enabling the gradual division of the sphincter muscle over time. This technique has demonstrated high safety and effectiveness, particularly for high-type anal fistulas, with consistently favorable outcomes.^9^ Seton therapy is also cost-effective compared to other treatments, such as fibrin glue, LIFT, and collagen plugs, although LIFT has demonstrated promising results.^8^

 The treatment of high anal fistulas is particularly challenging, as traditional laying-open procedures may sever a significant portion of the anal sphincter muscles, potentially leading to incontinence. To address this, various surgical techniques have been developed over time.^5^ Despite a growing body of literature on these techniques, there remains a need for comprehensive comparative studies to determine the most effective treatment options. This study aimed to compare the outcomes of cutting seton insertion and the LIFT procedure (preceded by draining seton insertion) in terms of healing time, recurrence rates, incidence of incontinence, and quality of life over a 5-year period.

## Materials and Methods

 This retrospective study included patients with high-type perianal fistulas who underwent LIFT or cutting seton insertion surgeries between May 16, 2018, and April 17, 2024, at Shahid Faghihi and Ghadir mother and child hospitals. The protocol for this study was approved by the Ethics Committee of Shiraz University of Medical Sciences, Shiraz, Iran (IR.SUMS.REC.1403.174). All patients provided informed consent to participate in the study.

###  Inclusion and Exclusion Criteria

 Adult patients ( > 18 years old) who had undergone surgery at least 3 months prior were included in the study, provided they met the following criteria:

- No history of inflammatory bowel disease, diabetes mellitus, malignancy, neurological disorders, other perianal diseases, or corticosteroid use.
- Willingness to participate in the study and complete the required follow-up assessments.

Exclusion criteria included patients with incomplete data, those lost to follow-up, or those with any conditions that could confound the study’s results (e.g., active infection at the time of the study).

###  Data Collection

 Medical records were used to gather demographic and clinical data, including body mass index (BMI), date of surgery, type of surgery, and duration of hospital stay post-surgery. Additional data—including chili pepper consumption, smoking habits, alcohol use, history of diarrhea and constipation, number of previous surgeries, duration of time spent sitting on the toilet, frequency of bowel movements, fecal incontinence, healing, and quality of life—were collected through telephone interviews with eligible patients.

 Fecal incontinence was assessed using the Wexner score,^10^ a validated tool that includes five questions about incontinence concerning solid, liquid, or gas, the need to wear a pad, and lifestyle changes. Scores for each question range from 0 to 4, with higher scores indicating worse fecal incontinence.

 Quality of life was assessed using the Short Form 12 Health Survey (SF-12),^11^ which evaluates both the physical component summary (PCS) and the mental component summary (MCS) based on eight health concepts: general health, physical functioning, role-physical, bodily pain, vitality, social functioning, role-emotional, and mental health.^12^

 Fistula healing was defined as the closure of the external opening of the fistula and the resolution of perianal pain and pus discharge within three months after surgery. Recurrence was defined as the reappearance of symptoms (pain, drainage, or external opening) after initial healing.^13^ Recurrence was further confirmed through clinical examination by a colorectal surgeon for patients who consented to follow-up at our clinic.

###  Surgical Procedures

*LIFT procedure (preceded by draining seton insertion):* Initially, a draining seton was inserted three to four weeks before the LIFT procedure to manage sepsis or active infection. Thereafter, the patient was placed in a prone position under spinal anesthesia. The external opening was inspected, and a proctoscopy was performed to identify the internal opening and the fistula tract. Hydrogen peroxide was injected into the external opening to confirm the internal opening’s location. A 1.5–2.0 cm curvilinear incision was made at the intersphincteric groove, and the fistula tract was dissected while preserving the anal sphincters. The intersphincteric tract was ligated using absorbable sutures (Vicryl 3/0), and the tract was divided. Infected tissue was removed, and the wound was loosely closed with interrupted, absorbable sutures. The external opening was left open for drainage.

*Cutting seton insertion:* Under spinal anesthesia, patients were positioned in the prone position. Methylene blue was injected through the external opening to identify the fistula tract and internal opening. A probe was passed, and an elastic cutting seton was inserted and tightened with 2-0 silk to apply gentle, continuous pressure. A fistulectomy was performed using electrocautery to open the tract, and the skin and subcutaneous tissue overlying the tract were incised. Hemostasis was achieved, and a sterile dressing was applied to the site. Postoperative management included careful monitoring and periodic tightening of the cutting seton every 2 weeks as needed.^14^

###  Postoperative Care

 For patients who underwent the LIFT procedure, a strict nothing by mouth (NPO) regimen was maintained for three days postoperatively. Intravenous antibiotics, including ceftriaxone and metronidazole, were administered. Patients were discharged with oral antibiotics and instructed to resume a regular diet after recovery.

 For patients undergoing cutting seton insertion, the same antibiotic regimen was prescribed, and the diet was resumed once recovery was achieved. Patients were discharged the day following surgery.

 All patients attended scheduled follow-up appointments, during which clinical assessments were conducted to monitor wound healing, infection, and recurrence.

###  Data Analysis

 Statistical analyses were performed using SPSS software version 25. Qualitative data are reported as frequency (percentage), while quantitative data are presented as mean ± standard deviation (SD) or median (interquartile range [IQR]), depending on the results of the Kolmogorov-Smirnov test. Comparisons between groups were conducted using Chi-square tests, Fisher’s exact tests, independent sample t-tests, or Mann-Whitney U tests. A *P* value of < 0.05 was considered statistically significant.

## Results

 Among the available medical records, nine patients were excluded from the LIFT group (one with quadriplegia, one with genetic disabilities, five who did not consent to participate, and two with incomplete medical records). Additionally, 18 patients were excluded from the cutting seton insertion group (seven who did not consent to participate, one due to death, five who were non-Persian, and five who were taking steroids). Finally, we included 51 patients who underwent LIFT surgery and 48 who received cutting seton insertion. The median duration of follow-up was 9.0 months (IQR: 5.0-13.0) in the LIFT group and 27.0 months (IQR: 13.0-45.0) in the cutting seton insertion group (*P* < 0.001). There were no significant differences between the two groups concerning demographic characteristics, behavioral factors, and postoperative fecal incontinence (Tables 1 and 2). However, patients who underwent the LIFT procedure were more likely to have a positive history of chronic constipation (*P* = 0.006) and previous surgery for anal fistula (*P* = 0.004) compared with those in the cutting seton group. Furthermore, patients who underwent LIFT had a lower BMI (*P* = 0.029) and were discharged from the hospital later than those in the seton group (*P* < 0.001) (Table 2). No statistically significant differences were observed between the two groups in terms of healing rates, recurrence, healing time, or quality of life (Table 3).

**Table 1:** Comparison of demographic characteristics between groups

**Variable**	**LIFT**	**Cutting seton insertion**	***P *** **value**
n (%)	51 (51.5)	48 (48.5)	
Sex, n (%)			0.183^†^
Male	41 (80.4)	33 (68.8)	
Female	10 (19.6)	15 (31.3)	
Ethnicity, n (%)			0.842^†^
Fars	36 (70.6)	33 (68.8)	
Others	15 (29.4)	15 (31.3)	
Marital status, n (%)			0.348^‡^
Single	4 (7.8)	7 (14.6)	
Married	47 (92.2)	41 (85.4)	
Education, n (%)			0.360^†^
Under diploma	17 (33.3)	17 (35.4)	
Diploma	18 (35.3)	11 (22.9)	
Academic degree	16 (31.4)	20 (41.7)	
Age at questionnaire completion (years), mean ± SD	47.69 ± 13.76	45.47 ± 10.44	0.397*
Age at surgery (years), mean ± SD	46.45 ± 13.81	43.72 ± 10.42	0.276*

Abbreviations: LIFT: Ligation of Intersphincteric Fistula Tract, SD: standard deviation. Between-group differences in variables were determined using an independent sample t-test (*) for parametric variables and a chi-square test (†) or Fisher’s exact test (‡) for categorical variables.

**Table 2:** Comparison of behavioral and medical features between the two groups

**Variable**	**LIFT**	**Cutting seton insertion**	***P*** ** value**
Chili pepper consumption, n (%)			0.078†
Never	8 (15.7)	17 (35.4)	
Usually	29 (56.9)	21 (43.8)	
Daily	14 (27.5)	10 (20.8)	
Current cigarette use, n (%)	6 (11.8)	8 (16.7)	0.484^†^
Current water pipe use, n (%)	10 (19.6)	7 (14.6)	0.508^†^
Alcohol intake, n (%)	4 (7.8)	4 (8.3)	> 0.999^‡^
History of chronic constipation, n (%)	24 (47.1)	10 (20.8)	**0.006** ^†^
History of chronic diarrhea, n (%)	3 (5.9)	1 (2.1)	0.618^‡^
Number of previous surgeries for anal fistula, n (%)			**0.004**^†^
0	19 (37.3)	32 (66.7)	
1	27 (52.9)	10 (20.8)	
≥ 2	5 (9.8)	6 (12.5)	
Time of sitting on toilet, n (%)			0.419^†^
< 3 minutes	19 (37.3)	24 (50.0)	
3-10 minutes	27 (52.9)	21 (43.8)	
> 10 minutes	5 (9.8)	3 (6.3)	
Wexner's fecal incontinence score, median (IQR)	0.00 (0.00-3.00)	0.00 (0.00-4.75)	0.269^¶^
Number of bowel movements/week, median (IQR)	14.00 (7.00-14.00)	14.00 (7.00-14.00)	0.183^¶^
Hospital discharge after surgery (day), median (IQR)	3.00 (2.00-4.00)	2.00 (1.00-3.00)	**<0.001**^¶^
Body mass index, mean ± SD	26.22 ± 3.46	27.96 ± 4.30	**0.029** ^*^

Abbreviations: LIFT: Ligation of Intersphincteric Fistula Tract; IQR: interquartile range, SD: standard deviation. Between-group differences were determined using an independent sample t-test for parametric variables (*), Mann-Whitney U test for non-parametric parameters (¶), and Chi-square (†) or Fisher exact (‡) tests for categorical variables. Bold denotes statistical significance (*P* value < 0.05).

**Table 3:** Comparison of healing, recurrence, and quality of life between two groups

**Variable**	**LIFT**	**Cutting Seton insertion**	***P*** ** value**
Healing/recurrence status, n (%)			0.364^†^
Healing	31 (60.8)	34 (70.8)	
Primary healing and subsequent recurrence	9 (17.6)	4 (8.3)	
Not healing	11 (21.6)	10 (20.8)	
Healing time (day), median (IQR)	30.00 (21.00-60.00)	60.00 (30.00-90.00)	0.085^¶^
Physical component score, median (IQR)	56.58 (41.00-56.99)	54.82 (49.42-56.76)	0.528^¶^
Mental component score, median (IQR)	55.25 (38.94-58.80)	54.40 (41.78-57.01)	0.633^¶^

Abbreviations: LIFT: Ligation of Intersphincteric Fistula Tract; IQR: interquartile range, SD: standard deviation. Between-group differences were determined using the Whitney U test for non-parametric parameters (¶) and the Chi-square (†) test for categorical variables.

## Discussion

 The findings of this retrospective cohort study provide insights into the comparative effectiveness of two surgical techniques, cutting seton insertion and LIFT, in managing high perianal fistulas. The outcomes of interest were not significantly different between the two procedures. Similarly, a systematic review and meta-analysis of 14 randomized controlled trials concluded that no single technique is definitively superior.^4^

 Despite our results, several studies suggest that LIFT may be a more effective option for expediting recovery. Mishra and colleagues^15^ noted that sphincter-preserving techniques like LIFT tend to facilitate faster healing (2–4 weeks). This is particularly important in the context of high anal fistulas, where fecal contamination of the wound can occur. According to a study by Dualim et al, the healing time for the LIFT procedure was 2 months, which was longer than that observed in our study.^16^ Conversely, the study conducted by Alapach and Khaimook^17^ reported a mean healing time of 2 weeks for the LIFT group, which was shorter than the healing time observed in our study. In our study, the mean healing time for cutting seton insertion was 60 days, significantly shorter than that reported by Patton et al (17.7 months).^18^ The literature concluded that improving healing rates hinged on careful patient selection. They strongly recommended controlling sepsis and accurately identifying secondary extensions and fistula tracts from the anal canal before attempting any repair.^19^ The practice of using a draining seton before the definitive procedure is becoming increasingly common in the management of anorectal fistulas. The LIFT procedure should be postponed if there is an active infection in the anorectal area. In such cases, a draining seton should be inserted for no more than 6–8 weeks before performing the LIFT procedure. Delaying the procedure for longer periods can make dissection more challenging during the LIFT.^20^

 The recurrence rates observed in our study suggest that cutting seton insertion may be superior to the LIFT procedure in preventing fistula recurrence (8.3% vs. 17.6%, not statistically significant). According to the study by Patton and colleagues, the recurrence rate with cutting seton insertion was 6.8%, which was lower than that observed in our study.^18^ On the other hand, in the study by Raslan and others, the recurrence rate after cutting seton insertion was 9.8% during the first year post-surgery, which was higher than that observed in our study.^21^ In our study, the recurrence rate after the LIFT procedure was 8.3%, contrasting with the 42% recurrence rate reported by Torre and others over an 18-month follow-up period.^22^ This marked difference may be attributed to their extended follow-up duration, which could more comprehensively report delayed recurrences. A systematic review in 2024 reported recurrence rates across multiple studies ranging from 2% to 43%,^23^ indicating that our study demonstrates a relatively low recurrence rate.

 In terms of quality of life, our study found no significant differences between patients undergoing the LIFT procedure and those undergoing cutting seton insertion. According to the study by Mylonakis and colleagues,^24^ patients who underwent anal fistula surgery did not experience an improvement in quality of life postoperatively. However, the study by Sun and others reported that quality of life after the LIFT procedure improved in areas such as lifestyle, coping, and depression.^25^ However, we lacked data on the preoperative quality of life of the subjects, which could have been compared with postoperative outcomes.

 It is important to note that significant differences in certain fundamental characteristics between the two groups of participants may introduce bias into the comparison of outcomes. Specifically, patients in the IFT group reported a higher rate of constipation and more previous surgeries for anal fistula. Post-surgical persistent constipation may further increase the risk of trauma and sphincter hypertonia, leading to prolonged healing times and elevated recurrence rates.^26^ Additionally, prior anorectal surgeries can result in alterations to sphincter anatomy or function, making subsequent surgeries more technically challenging, impairing postoperative healing, and increasing the likelihood of recurrence.^27^ Therefore, these risk factors among patients who underwent the LIFT procedure, along with their lower BMI, may complicate the comparison of outcomes between these two groups.

 Our study had several limitations. The median duration of follow-up was 9 months in the LIFT group, which was significantly shorter than that of the other group and may not be sufficient to capture the true complication rate, potentially biasing the results in its favor. Furthermore, the small sample size is another limitation of the current study. Further studies addressing these limitations are highly recommended to enable a more comprehensive evaluation of these procedures.

## Conclusion

 Our study demonstrates no significant difference between the LIFT procedure (preceded by draining seton insertion and cutting seton insertion in terms of healing time, recurrence rate, incontinence, and quality of life. The selection of treatment should take into account the type of fistula, the patient’s symptoms, and the desired outcomes.
